# Development of a stochastic agent-based model to evaluate surveillance strategies for detection of emergent porcine reproductive and respiratory syndrome strains

**DOI:** 10.1186/s12917-017-1091-7

**Published:** 2017-06-12

**Authors:** A. G. Arruda, Z. Poljak, D. Knowles, A. McLean

**Affiliations:** 1 Department of Population Medicine, Ontario Veterinary College, University of Guelph, 50 Stone Rd East, Guelph, ON N1G 2W1 Canada; 2 Department of Computer Science, Computational Epidemiology and Public Health Informatics Lab, University of Saskatchewan, 176 Thorvaldson Bldg, 110 Science Place, Saskatoon, SK S7N 5C9 Canada

**Keywords:** Stochastic agent-based model, Porcine reproductive and respiratory syndrome control, Porcine reproductive and respiratory syndrome surveillance, Risk-based surveillance, Surveillance system sensitivity

## Abstract

**Background:**

The objective of the current study was to develop a stochastic agent-based model using empirical data from Ontario (Canada) swine sites in order to evaluate different surveillance strategies for detection of emerging porcine reproductive and respiratory syndrome virus (PRRSV) strains at the regional level. Four strategies were evaluated, including (i) random sampling of fixed numbers of swine sites monthly; (ii) risk-based sampling of fixed numbers, specifically of breeding sites (high-consequence sites); (iii) risk-based sampling of fixed numbers of low biosecurity sites (high-risk); and (iv) risk-based sampling of breeding sites that are characterized as low biosecurity sites (high-risk/high-consequence). The model simulated transmission of a hypothetical emerging PRRSV strain between swine sites through three important industry networks (production system, truck and feed networks) while considering sites’ underlying immunity due to past or recent exposure to heterologous PRRSV strains, as well as demographic, geographic and biosecurity-related PRRS risk factors. Outcomes of interest included surveillance system sensitivity and time to detection of the three first cases over a period of approximately three years.

**Results:**

Surveillance system sensitivities were low and time to detection of three first cases was long across all examined scenarios.

**Conclusion:**

Traditional modes of implementing high-risk and high-consequence risk-based surveillance based on site’s static characteristics do not appear to substantially improve surveillance system sensitivity. Novel strategies need to be developed and considered for rapid detection of this and other emerging swine infectious diseases. None of the four strategies compared herein appeared optimal for early detection of an emerging PPRSV strain at the regional level considering model assumptions, the underlying population of interest, and absence of other forms of surveillance.

## Background

Porcine reproductive and respiratory syndrome (PRRS) is an endemic infectious swine disease caused by an RNA virus and is responsible for considerable economic losses in North America and many European countries [1, 2]. The syndrome is characterized by decreased growth in pigs across all ages (mainly due to respiratory disease and secondary infections) and reproductive failure in adult female pigs. Even though strategies for PRRS control and elimination have been previously investigated and described [3], it remains a challenge for the swine industry. Factors related to the current characteristics of the North American swine industry contribute to the maintenance of PRRS within a country or region. Among these are the high degree of connectivity between swine sites, the constant exchange of subpopulations of animals that are at higher risk for disease transmission between sites (e.g. weaned piglets and culled sows), the high turnover rates of animals within farms, and the segregated nature of the different phases of production [4].

Currently, in Canada, the most commonly used approach for PRRSV surveillance is the submission of specimens from suspected clinical cases to diagnostic laboratories. This is commonly complemented by other activities that include: (i) detection of PRRSV cases through on-going monitoring when expected prevalence is low (e.g., nursery sites from specific production systems), (ii) certification of absence of infection using minimum pre-specified level, or (iii) specific regional studies or programs conducted to assess trends in disease prevalence or incidence over time. With the advent of regional disease control programs for PRRS, a frequently posed question relates to the design of effective surveillance strategies when one of the objectives is detection of new cases due to circulation of a novel PRRSV strain. In this context, risk-based surveillance strategies have been widely used and justified as effective and efficient strategies. The main idea behind this approach is that it targets subpopulations of animals that are at increased risk for the occurrence of infection due to the presence of known risk factors [5]. Although implementation of risk-based approaches to sampling in food animal veterinary medicine is relatively frequent under field conditions, its quantitative assessment has been relatively limited to cases of supporting declaration of freedom from infection in a jurisdiction [6], or for comparisons between alternative surveillance strategies before a recommendation is made [7]. Typically, assessment of surveillance systems has been accomplished using stochastic scenario tree modelling (STM). This methodology models the process of disease detection while including factors that affect probability of infection or detection of a surveillance system [8].

Risk-based approaches could be considered when detection of emerging strains of an endemic pathogen is important. However, the framework for the quantitative assessment of alternative surveillance approaches for such situation is not well described in the relevant literature. Velasova et al. [9] used a stochastic STM approach to evaluate the expected performance of a passive monitoring system for detection of novel strains of PRRSV in the United Kingdom. Even though STM appears a logical extension when the main objective is detection of cases due to circulation of a novel PRRSV strain for endemic situations, its uniform application in all situations is limited due to two main reasons. Firstly, the goals and approaches to surveillance within endemic scenarios could vary greatly (e.g. monitor disease trends over time, detecting new cases and possibly novel pathogen strains, etc.), and secondly the modern swine production systems are hierarchically structured and networks have been identified as important contributors to disease spread [10, 11], representing yet another layer of risk factors that would be difficult if not impossible to incorporate using STM. These risk factors would be difficult to incorporate in a typical STM, and an alternative is to develop agent-based models (ABM) with swine sites as agents. Such an approach is particularly appealing in the context of disease control projects because observed data on risk factors can be easily incorporated at the site level, together with contact structure among swine sites. In addition, performance of high-risk surveillance strategies could be compared to high-consequence surveillance strategies in a natural manner, since the directed flow of infected animals could be incorporated in such models explicitly [12].

The goal of the current study was to develop a stochastic ABM that would allow for the evaluation of different surveillance strategies for detection of emerging PRRSV strains at the regional level. Four surveillance strategies were evaluated, including (i) random sampling of fixed numbers of swine sites monthly; (ii) risk-based sampling of fixed numbers of specifically breeding swine sites (high-consequence sites); (iii) risk-based sampling of fixed numbers of low biosecurity sites (high-risk sites); and (iv) risk-based sampling of breeding sites that are also characterized as low biosecurity sites (high-risk/high-consequence sites). The main outcome of interest was the sensitivity of the surveillance systems evaluated, i.e. the probability of the systems in detecting infected sites. Furthermore, the time to detection of the three first cases was also described, and sensitivity analysis was conducted to evaluate the impact of the target design prevalence (level of disease that the system aims to detect) used for sample size calculation (1%, 2% or 5%) in the main outcomes of interest.

## Methods

A stochastic agent-based model was created and implemented using the software Anylogic® version 7.1.2 (XJ Technologies, St Petersburg, Russia). The model description outlined in the next paragraphs follows general reporting guidelines from the standardized overview, design concepts, and details (ODD) protocol as described by Grimm et al. [13].

### Purpose

The main purpose of the model was to simulate the dynamics of a representative sample of Ontario swine sites and the spread of PRRSV between these sites using empirical data from the PRRS Area Regional Control and Elimination (ARC&E) projects in Ontario, Canada. The model would allow for the evaluation of regional PRRS surveillance strategies to detect emerging PRRS virus cases within this population of interest.

### State variables and scales

The model features a collection of swine sites that can transfer PRRSV infections through their trucking, production system, and feed networks. A swine site was defined as one or more barns built close together that housed pigs in a specific physical location. A total of 816 swine sites were enrolled in a provincial PRRS control project when this study was conducted and this corresponded to our population of interest.

Swine sites were given characteristics described by parameters, which were extracted from the Ontario Swine Health Advisory Board regional control program database from a standardized questionnaire answered by swine producers as they enrolled in the project. This database was privately owned and maintained by the Ontario Swine Health Advisory Board, and the authors were given permission to access it by this entity. These parameters were modelled as being static, and therefore were not allowed to change over time. Characteristics included were animal flow (continuous animal flow or all-in all-out animal flow), number of neighbors (categorized into zero neighbors, one to five neighbors, and more than five neighbors), presence of a shower in facility (yes or no), number of animals (categorized into up to 500 animals, from 500 to 2000 animals, and more than 2000 animals), and production type (breeding site, nursery site, and growing pig site, the last included wean-to-finish and finishing operations). For a relatively small percentage of sites, the information regarding one or more parameters was missing, and for those the model was set to randomly assign a category for the parameters. This occurred for 85 sites (10%) for the parameter ‘presence of shower facility’, 23 sites (3%) for number of animals, and 217 sites (27%) for animal flow. These characteristics were selected because they have been previously reported as risk factors for PRRS [14, 15] and because the information was readily available from the control program.

The increase or decrease in the risk of PRRSV infection according to those risk factors was specified at model start according to values found in Table 1. At receipt of an ‘exposure’, a non-infected swine site would become infected with a 10% probability. This was then multiplied by the relative risk of infection factors relevant to the model, animal flow, number of neighboring facilities, presence of showers, number of animals at the facility, and immunity status (Table 1).

**Table 1:** Definition of parameters and values used for model simulations

Parameter	Value (unit)
Time to PRRSV^a^ elimination for breeding sites	385 (days)^b^
Time to PRRSV elimination for AIAO^c^nurseries	56 (days)
Time to PRRSV elimination for AIAO finishers/ wean-to-finish sites	112 (days)
Baseline probability of infection with PRRSV (new strain)	10% per year
Percent of the swine sites considered “completely susceptible”	63%^d^
Relative Risks for getting infected with PRRSV	
Relative risk of getting infected with PRRSV for nurseries compared to breeding sites	1.2
Relative risk of getting infected with PRRSV for finishers/ wean-to-finish sites compared to breeding sites	1.5
Relative risk of getting infected with PRRSV for sites with no shower in facility compared to sites with shower	1.3
Relative risk of getting infected with PRRSV for sites with continuous flow compared to sites with AIAO	1.5
Relative risk of getting infected with PRRSV for sites with medium number of neighbours compared to sites with no neighbours	1.5
Relative risk of getting infected with PRRSV for sites with high number of neighbours compared to sites with no neighbours	2.0
Relative risk of getting infected with PRRSV for sites with medium number of animals compared to sites with reduced number of animals	1.5
Relative risk of getting infected with PRRSV for sites with high number of animals compared to sites with reduced number of animals	2.0
Relative risk of getting infected with PRRSV for naïve sites compared to sites with complete immunity	2.0
Relative risk of getting infected with PRRSV for sites infected with other PRRSV strains (partial immunity) compared to sites with complete immunity	1.5

Formula for probability of infection

0.10^∗^ (relative risk [RR]) animal flow^∗^ (RR) number of neighbors^∗^ (RR) presence of shower^∗^ (RR) number of animals^∗^ (RR) production type^∗^ (RR) immunity status

^a^Porcine reproductive and respiratory syndrome virus

^b^Linhares et al., 2014 [21]

^c^All-in, all-out animal flow

^d^Arruda et al., 2015 [22]

**Fig. 1:**
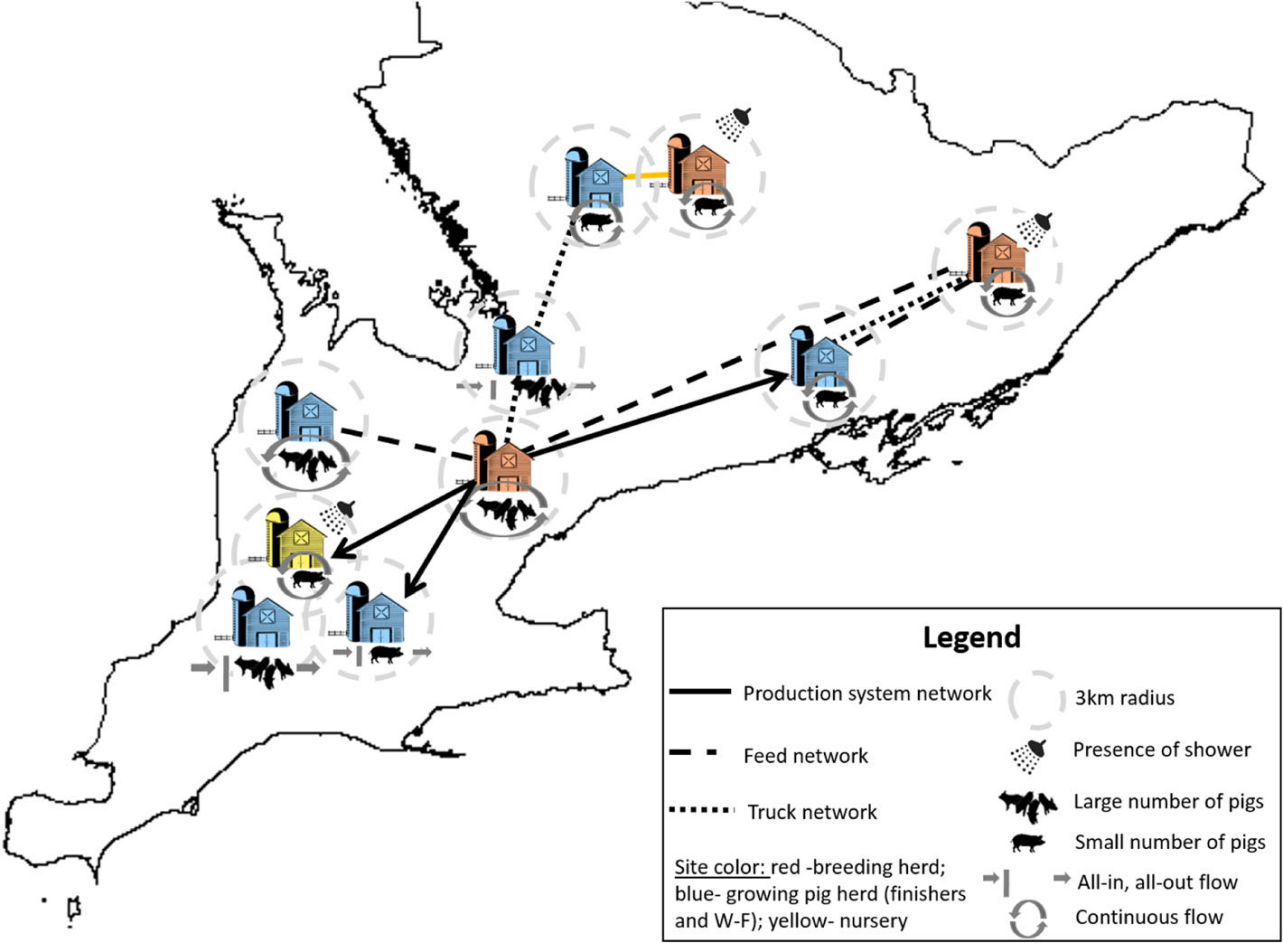
Model scheme using ten hypothetical swine sites characteristics and locations in Southern Ontario. Underlying immunity of swine sites are not shown due to their dynamic nature. A link between swine sites represented a common service provider within the specific network

Swine sites were eligible to be part of up to three different static undirected networks: production system, truck, and feed networks. Sites were considered connected through each of these networks if the swine producer (site owner) had named a common ownership structure (for production system), a common transportation company (for the truck network), and a common feed company (for the feed network) as other swine producer(s). This information was collected during administration of the same questionnaire previously mentioned and corresponded to ‘static’ relationships (no frequency of contact information collected). A simplified visualization of the model with site characteristics and network connections is shown in Fig. 1.

The model proceeded in daily time steps, with the new PRRSV strain introduced on day 3 after model start and a follow-up time of approximately 2 years and 8 months (1000 days). It was assumed that this population of 816 swine sites was stable for the time evaluated, with no swine sites being added or removed from the population.

### Process overview and scheduling

**Fig. 2:**
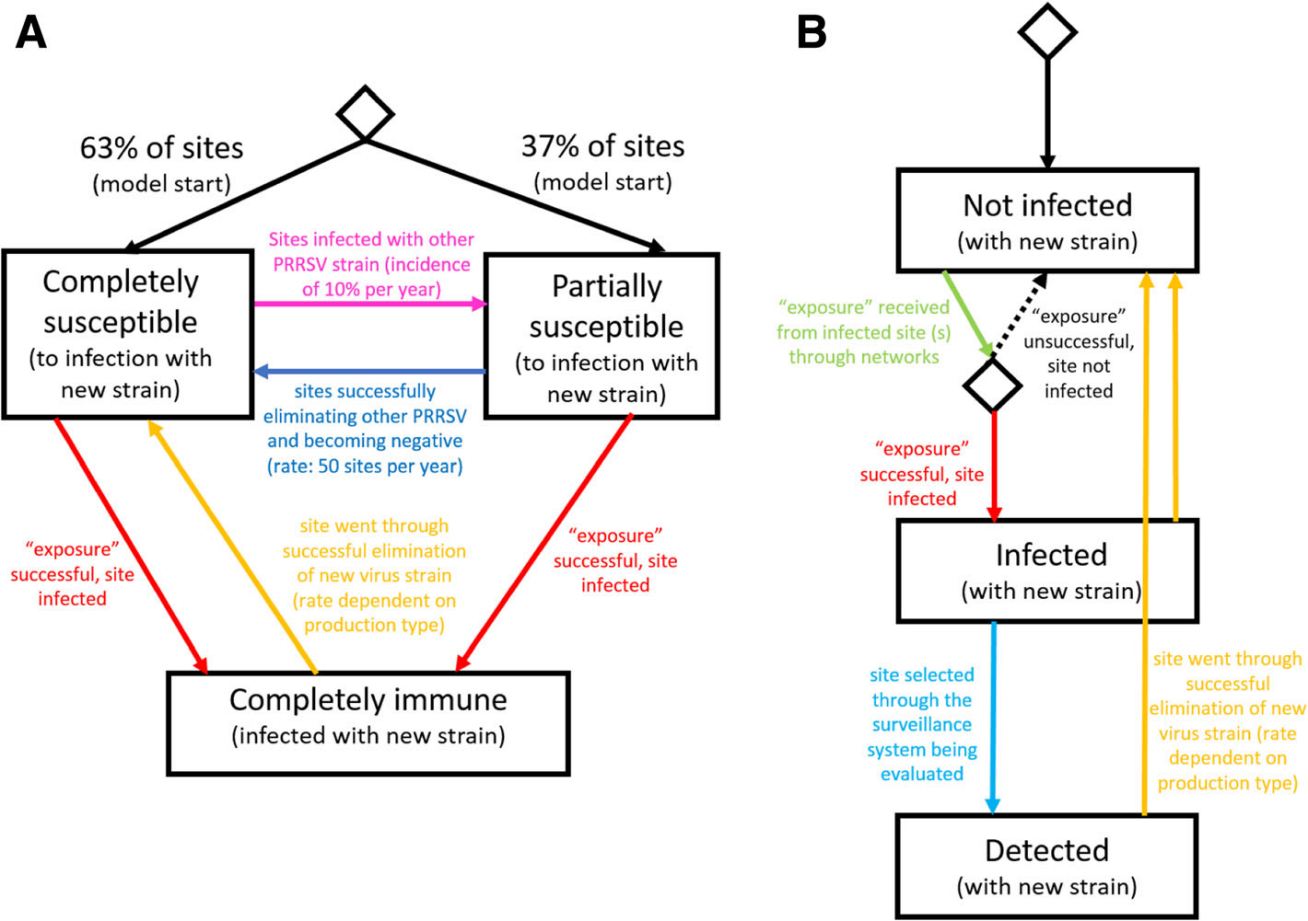
Compartmental states for swine sites. **a**. Underlying immunity state chart considering infection with other porcine reproductive and respiratory syndrome virus (PRRSV) strains; and **b**. New infections and new detections state chart

Swine sites included in this model were characterized by mutually exclusive PRRS immunological status that included ‘completely susceptible’, ‘partially susceptible’, and ‘completely immune’. The nomenclature given to this so-called ‘immunity status’ refers to the likelihood of infection with the new virus strain that was purposely introduced as a ‘challenge’ in order to evaluate the surveillance strategies. Detailed rules for such classification are presented in Table 2. In summary, swine sites within the ‘completely susceptible’ compartment corresponded to sites containing a naïve animal population, characterized by being both seronegative and virus negative, therefore reflective of no previous or current PRRSV exposure. Sites within the ‘partially susceptible’ compartment corresponded to swine sites that were serologically positive or positive by virus detection, which meant that the population of animals within these sites was at a lower risk for infection with a new PRRSV strain compared to the naïve population due to partial immunity conferred by antibodies produced against a heterologous PRRSV strain. As a proportion of swine sites may choose to eliminate PRRS viruses from their herds (i.e. move from the compartment ‘partially susceptible’ to ‘completely susceptible’), and as a proportion of negative sites get infected yearly according to a baseline PRRS incidence rate (i.e. move from the compartment ‘completely susceptible’ to ‘partially susceptible’), immunity statuses were allowed to change over time (Fig. 2a).

**Table 2:** Definition of site immune status according to the PRRS control program and model assumptions for underlying immunity

				Breeding sites	Growing pig sites
Immunity level^a^	OSHAB classification	Serology^b^	Virus detection^c^	Comments	Comments
Completely susceptible	Confirmed negative	Negative	Negative	Series testing	Series testing
Partially susceptible	Confirmed positive	At least one positive		Parallel testing	Parallel testing
Completely susceptible	Presumed negative	-	-	Sample size not met, sample of downstream growing pig sites	Sample size not met, sample of upstream sow sites
Partially Susceptible	Presumed positive	-	-	Downstream sites confirmed positive by diagnostic test, veterinarian assessment of site	Upstream sites confirmed positive by diagnostic test, veterinarian assessment of site

^a^As defined by the current model (underlying swine site immunity)

^b^Evidence of previous exposure to porcine reproductive and respiratory syndrome virus (PRRSV), measured through ELISA testing for antibody detection in serum or oral fluids

^c^Evidence of current virus infection, measured via PCR testing in serum, oral fluids or tissue samples

At model start, none of the sites were classified as ‘completely immune’ because it was assumed that the virus introduced into the population was completely new to the population of animals within sites. Swine sites would transition into that immunological compartment if animals within those sites were infected with the new virus strain as model progressed. In this case, it was assumed that a sufficient percentage of animals were exposed to the new virus and sufficiently protected so that the site was considered ‘completely immune’ for a limited period of time, and therefore could not get re-infected while in this compartment. Sites could also opt for and successfully eliminate the virus and return to the ‘completely susceptible’ compartment at rates that varied according to production type (Figure 2a; Table 1).

### Design concepts

Interactions between agents (indirect contacts among swine sites) were modeled using the three above-mentioned networks. At model start, networks were loaded in Anylogic® as symmetric matrices extracted from network analysis using UCINET 6 [16]. Each of the three matrices contained site identifiers and “1” in case the two sites had an indirect contact through the specific network, and “0” otherwise. Networks were represented separately, and the frequency of contact between swine sites through these networks was considered more intense for the production system network (a contact was assumed to occur one time per week), followed by the truck network (one contact every two weeks) and the feed network (one contact per month). The role of the production system network was to reflect the movement of people, personnel, equipment, and animals between sites, and two sites that were linked through this network could be connected directly or indirectly, depending on the nature of the movement (considering site type). For the truck and feed networks, however, connections between sites were always indirect. Due to the type of service involved (movement of genetic stock, deadstock, slaughterhouse transportation, besides others for the truck network; and delivery of feed for the feed network), it was assumed that the truck network would be heavier in the “disease transmission scale” compared to the feed network.

Transmission of disease between sites was modelled through communications via the use of Anylogic© ‘messages’ reflecting exposure to the virus (opportunity for infection) that could result in infection or not. The virus was introduced into the population of sites through random selection of one site in the population to be infected. Following this, the infected site would expose other sites within its networks at the frequencies established above. At receipt of an ‘exposure’, non-infected swine site’s characteristics were considered and a stochastic process was carried out using the formula on Table 1 to determine whether a site would be infected or not. This described process was modelled using compartments within a state chart as shown in Fig. 2b. It is important to note that, uniquely for the production system network, the direction of ‘exposure’ was taken into account. The production type for the site being the source of the ‘message’ (the infected site exposing others, or the sender of the ‘message’) was assessed, as well as the production type of site that was receiving the exposure (site at risk for infection, or receiver of the ‘message’). In cases where the exposure was between two sites of the same production type, the risk of infection was considered relatively small, since it would be most commonly reflective of indirect transmission through sharing of site personnel, equipment, etc. On the other hand, when the direction of exposure was from a breeding herd to a nursery or growing pig operation, as well as if it was from a nursery to a growing pig operation, the likelihood of infection was assumed to be higher because there could be movement of animals in such direction, which would be indicative of direct PRRSV transmission. The comparison between those numbers can be obtained in Table 1. Finally, it was assumed that swine sites had one outbreak every 20 years due to modes of transmission non-specified in this model (including but not limited to airborne transmission).

### Initialization

At model start, surveillance system screenings were set to occur through Anylogic© ‘events’, triggered at regular intervals (monthly, or every 30 days) starting on day zero. The number of swine sites to be checked (inspected) for infection with the emerging PRRSV depended on the pre-determined design prevalence (Table 3).

**Table 3:** Description of porcine reproductive and respiratory syndrome site-level surveillance scenarios investigated

Scenario^a^	Type of sampling	Design prevalence (number of sites sampled)
Random_1	Random	1% (23)
Random_2	Random	2% (13)
Random_5	Random	5% (5)
HR_1	Risk based - High-risk^b^	1% (23)
HR_2	Risk-based - High-risk^b^	2% (13)
HR_5	Risk-based - High-risk^b^	5% (5)
HC_1	Risk-based - High-consequence^c^	1% (23)
HC_2	Risk-based - High-consequence^c^	2% (13)
HC_5	Risk-based - High-consequence^c^	5% (5)
HR/HC_1	Risk-based - High-risk and high-consequence^d^	1% (23)
HR/HC_2	Risk-based - High-risk and high-consequence^d^	2% (13)
HR/HC_5	Risk-based - High-risk and high-consequence^d^	5% (5)

^a^Each scenario consisted of 1000 simulations

^b^High-risk based sampling consisted of sampling of swine sites that were considered at the highest risk of being infected: sites that had low biosecurity (no shower-in facility and continuous animal flow)

^c^High-consequence based sampling consisted of sampling of swine sites that were considered at the highest risk of infecting other sites: breeding sites

^d^High-risk and high-consequence based sampling consisted of sampling of swine sites that had a combination of the highest risk of getting infected as well as the highest risk of infecting others: breeding herds that had low biosecurity (no shower-in facility and continuous animal flow) herds

The evaluation of each regional surveillance strategy began after the challenge-virus introduction on day 3 for each simulation. The model ran for three days before virus introduction to assure loading of network and parameters datasets.

### Inputs

Model inputs were obtained from the peer-reviewed literature whenever possible, and when not available, they were obtained from discussions with experts in the area of swine production. A list of inputs used in the model is shown in Table 1.

### Model calibration and statistical analysis of outcomes

The model was calibrated to reproduce plausible values in terms of the underlying immunological status of the population for an endemic disease such as PRRS in North America. The aim was to produce a mean PRRS prevalence between 30 and 40% within the two-year period evaluated. There is no available information to the knowledge of the authors concerning the quantification of system sensitivity for any surveillance strategy; therefore, it was unfeasible to compare current model results with expected outcomes. The outcomes measured herein included daily total number of sites that were infected with the new PRRS virus strain, total number of infected sites that were detected and total number of non-infected sites. Data processing was conducted using Excel® (2012, Microsoft Corporation) and Stata 13 (Stata-IC version 13; StataCorp, 2007, College Station, Texas, USA); descriptive analyses, surveillance sensitivities, Kaplan-Meier survival functions and median survival times were conducted and calculated using Stata 13. For the construction of survival curves, only simulations that had at least three cases were eligible to have the outcome of interest (time to detection of first three cases). The median survival time was compared across scenarios, which refers to the time by which 50% of the eligible simulations (simulations with at least three cases) achieved detection of a minimum number of three cases.

### Simulation experiments

A total of 1000 simulations were run for each of the evaluated scenarios using a random seed. Twelve scenarios were investigated, as described on Table 3. Site surveillance was programmed through cyclic events. Four main scenarios were investigated that are detailed in the following paragraphs, and the fixed number of swine sites to be sampled varied according to the desired design prevalence: 1% (*n* = 23 sites per month), 2% (*n* = 13 sites per month), or 5% (*n* = five sites per month). Sample sizes were calculated with the objective of demonstrating freedom of infection at the population level, and were calculated using the online tool “FreeCalc” (AusVet Animal Health Services©) [17] considering 100% herd level specificity, 95% herd level sensitivity, type I error of 0.05, a population size of 816 and the modified hypergeometric exact calculation method. Sample sizes were calculated using annual specified design prevalence, and then the total number to be sampled was divided by 12 to give the monthly number of sites to be sampled. For all scenarios, it was assumed that, even when a swine site was detected with the new PRRSV strain, transmission to other sites was still possible which is reflective of field conditions for PRRSV and other endemic viral pathogens. It was further assumed that sampling size requirements for detection of PRRSV within herds was met in all cases (no false positives among sites selected for sampling and sufficient number of animals sampled to detect pre-specified within herd design prevalence).

#### Baseline scenario

The baseline scenario consisted of random sampling a fixed number of swine sites each month (varying according to desired design prevalence), based on sampling with replacement (swine sites were eligible for selection in the next sampling event, unless they were detected).

#### High-risk scenario

The high-risk scenario targeted as sampling units swine sites that had low biosecurity measures: a combination of both continuous animal flow and the absence of a shower facility in the site. The sampling consisted of a random sampling of fixed numbers of swine sites (varying according to desired design prevalence) that met both criteria described above (*n* = 342, number is approximate due to the fact that sites with missing information were randomly assigned to an equal distribution of the possible responses).

#### High-consequence scenario

The high-consequence scenario targeted as sampling units swine sites that were considered to have high consequence if infected, i.e. they could potentially spread disease to multiple sites. This population of sites corresponded to breeding herds (*n* = 259).

#### High-risk/ high-consequence scenario

The final scenario corresponded to the targeted sampling of sites that met both high-risk/high-consequence criteria, therefore the eligible pool were sites that were breeding herds with low biosecurity (both absence of a shower facility and continuous animal flow), number of eligible sites varied from 62 to 157, depending on how sites with missing information were assigned to characteristics during model runs). For all evaluated scenarios, once sites were detected as infected by the surveillance system, they were excluded from the pool of sites eligible for selection.

## Results

**Fig. 3:**
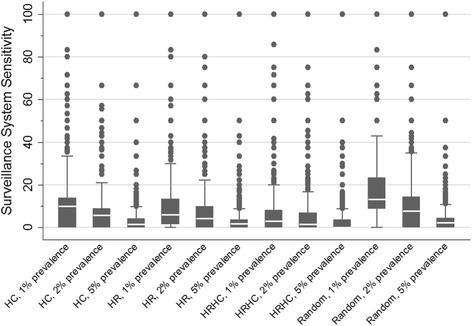
Surveillance sensitivity boxplots for the total of simulations, by surveillance strategy. HC: high consequence, HR: high risk, HRHC: high risk- high consequence

Results from the model were that, under the conditions specified, all evaluated surveillance strategies showed relatively low overall mean sensitivity in detecting an emergent PRRSV strain over an approximate three-year period (Table 4). It is, however, important to note that the surveillance sensitivity distribution across simulations within the different surveillance strategies was highly right-skewed, with a small number of simulations yielding high sensitivity for almost all strategies (Fig. 3); these cases were in majority cases in which a small number of sites were infected. The number of simulations at which the mean surveillance system sensitivity and the variance stabilized varied according to scenario, but approximately after 100 simulations this happened across all scenarios (data not shown).

**Table 4:** Mean (%), standard deviation (%), minimum (%) and maximum (%) surveillance system sensitivities; and median total of infected sites according to surveillance scenarios evaluated

Scenario^a^	Surveillance system sensitivity^b^				Median of total infected sites (IQR)^c^
	Mean	SD	Minimum	Maximum	
Random_1	17.6	21.7	0	100.0	26.0 (100.0)
Random_2	7.5	11.3	0	50.0	18.0 (69.0)
Random_5	10.7	25.9	0	100.0	21.0 (101.0)
HR_1	14.1	23.4	0	100.0	24.0 (101.5)
HR_2	18.2	30.8	0	100.0	22.0 (100.0)
HR_5	6.9	19.5	0	100.0	24.0 (100.0)
HC_1	20.4	29.4	0	100.0	26.0 (101.0)
HC_2	7.9	18.3	0	100.0	24.0 (101.0)
HC_5	3.6	5.3	0	23.0	23.0 (139.0)
HR/HR_1	7.2	18.2	0	100.0	20.0 (101.0)
HR/HC_2	7.9	18.4	0	100.0	18.0 (94.0)
HR/HC_5	9.9	24.9	0	100.0	24.0 (100.0)

^a^Each scenario consisted of 1000 simulations; please refer to Table 3 for detailed scenario definitions

^b^Calculated as the fraction of number of cases detected divided by the total of infected cases, for each simulation, considering in a period of approximately three years

^c^Interquartile range

As expected, for all scenarios, as design prevalence decreased (and number of sites sampled per month increased), system sensitivity also tended to increase, which is reasonable given the increase in the likelihood of sampling infected sites. Interestingly, this observation was not evident for the high-risk or high-risk/ high-consequence scenarios (Table 4 and Fig. 3).

**Fig. 4:**
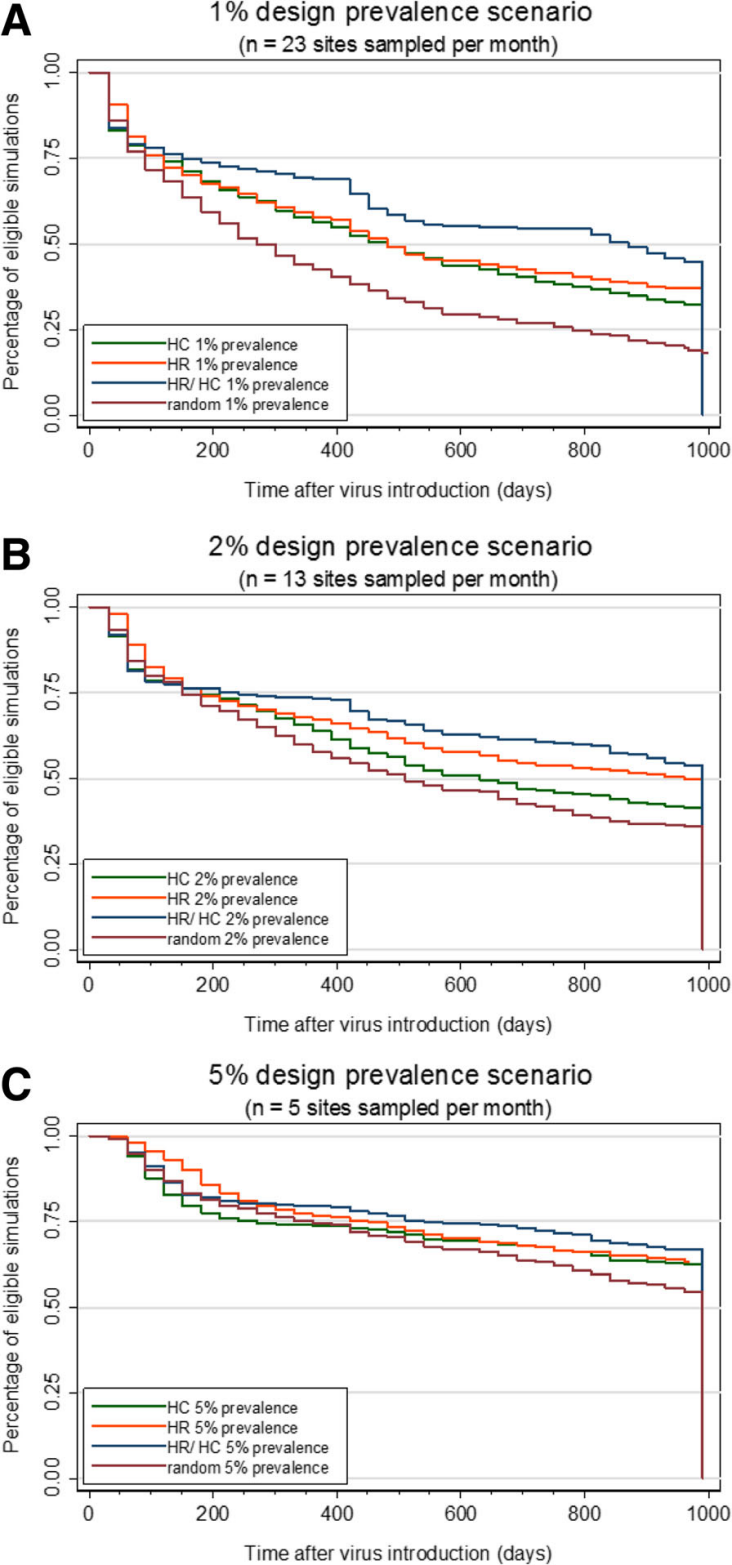
Kaplan-Meier survival functions showing time to detection of first three cases; according to different sample sizes calculated for 1% (**a**), 2% (**b**) and 5% (**c**) design prevalence, and stratified by surveillance strategies. HC: high consequence, HR: high risk, HR/HC: high risk- high consequence. Only simulations with at least three infected sites were eligible for this outcome

Kaplan-Meier survival functions showed that, regardless of design prevalence, the random sampling surveillance strategy was the one for which detection of the three first cases was faster over an approximate time period of three years when compared to the other strategies (Fig. 4).

Under the 1% design prevalence scenario, median survival time was 271 days (SE: 18.79) for the random sampling strategy and 481 days for both the high-consequence (SE: 30.87) and high-risk (SE: 27.85) scenarios. For the 2% prevalence scenario, the median survival time was 511 days (SE: 42.19) for the random sampling strategy, 631 days (SE: 53.74) for the high-consequence scenario, and 961 days (SE: 11.00) for the high-risk scenario. The median survival time was not reached for the 5% prevalence scenario, regardless of surveillance strategy examined. The high-risk/ high-consequence strategy only reached a median survival time under the 1% prevalence scenario, and this point was reached in 871 days (SE: 68.92).

## Discussion

The model developed herein simulates transmission of a hypothetical emerging PRRSV strain between swine sites through three important industry networks; production system, truck and feed networks. Importantly, this model considers sites’ underlying immunity due to past or recent exposure to heterologous PRRSV strains, as well as the different likelihood of infection due to previously described demographic, geographic and biosecurity-related PRRS risk factors [14, 15]. The present study fulfilled its main objective of development of a tool for the evaluation of surveillance systems for situations where emergent cases (e.g. emerging genotype) of a certain disease are to be detected within a specific area in which the disease is already endemic; in such the emphasis of the system should be on timely detection of new cases [18]. Many times such evaluation is both financially and logistically unfeasible to complete under field conditions.

While there are numerous arguments in favor of risk-based sampling for disease detection [19], the low surveillance system sensitivities found for all the risk-based sampling scenarios (as implemented in this study), was not unexpected considering what has been previously reported on the occurrence of PRRS within the Ontario swine industry. Previous work has shown that given the unique nature of such an industry, focusing on demographics or biosecurity characteristics of individual sites for risk-based surveillance would not yield the most impactful strategies because the most important determinant of PRRS status has been reported to be the production system and not site characteristics on their own [11].

The authors suggest that the target of risk-based sampling needs to be reconsidered and strategies need to be developed considering how production systems are connected, the importance of sites in the different networks, and the number of sites within network components. Additionally, it would be worthwhile to evaluate the manner by which downstream site status should be handled in cases where breeding sites are detected as infected. The current approach for PRRS ARC&E projects in Ontario is to automatically declare downstream sites from positive breeding herds as positive by animal flow, a measure that can result in false positive classifications. An alternative would be to prioritize sampling of downstream growing pig sites, action which could result in rapid depletion of resources in cases breeding sites from large production systems are infected. These additional scenarios were not evaluated herein, but are examples of a future research area that is very applicable under field conditions. To the best knowledge of the authors, there is no available information on quantification of active surveillance system sensitivity for PRRS in endemic disease contexts that would allow for comparison to current study results.

In general, all survival times (time to detection of the first three cases) for examined scenarios were long. We therefore conclude that none of the compared strategies were optimal for early detection of this disease given model assumptions, the underlying population, and absence of other forms of surveillance. The median survival time was never reached for the 5% design prevalence scenario, which supports the fact that, regardless of the strategy examined, if sample size is limited, prompt detection of potential outbreak cases will very likely not occur (considering such sampling is the only strategy in place to detect new infection cases).

There are important limitations to the current study that need to be acknowledged. Firstly, data were obtained from a limited portion of the Ontario swine industry (estimated at approximately 30% of all sites), and therefore the service providers network is not complete. Even though there is no reason to believe that the sample of sites are selectively biased in any form, the possibility exists that connections from absent sites could potentially change the structure in meaningful ways, and that could impact disease spread in unpredictable ways. Given this limitation, it is important to re-state that the focus of this study was primarily the development of the methodology and the comparison among surveillance strategies themselves. Another important issue is the lack of data for PRRS parameter estimations such as the baseline probability of infection given exposure and relative risks presented on Table 1, as well as the fact that authors relied on expert opinion for the selection of risk factors for disease introduction (e.g. animal flow and number of neighbors). It is important to consider these assumptions when interpreting results of the model.

Furthermore, passive surveillance systems were not taken into consideration in the current study, and in the case where the challenge PRRSV was a highly virulent strain, the role of passive surveillance might have been particularly important and increased disease recognition and control efforts. However, sensitivity of passive surveillance is difficult to estimate. In England, the probability of infected farms being detected through passive surveillance for PRRS was reported as low, with a mode of 7.4% assuming 35% active PRRS infection prevalence [9]. This varied when regional pig density and use of vaccination were considered, with farms in a low pig density area and not using a vaccine having the lowest detection probability. Sensitivity analysis conducted in that study showed that an important parameter, as expected, was the probability that an infected pig would show clinical signs [9]. In a different context, during detection of the PRRS outbreak in Sweden in 2007 [20], active surveillance had a major role, and that particular outbreak was detected from the annual surveillance program and not due to clinical suspicion, even though animals from the whole country were naïve to the virus. The authors of the current study were interested not in quantifying the passive surveillance system per se, but in evaluating which of other active surveillance strategies would optimize disease detection.

An important assumption of the model was that transmission was possible even after swine sites were detected as infected by the surveillance systems. The authors acknowledge that this is to a certain extent contradictory to the core intention of surveillance for early detection, but argue that it is a plausible assumption given the current structure of this dynamic industry. Most of the time it is simply logistically impossible to stop animal movement or coordinate and schedule service providers according to PRRS status in a timely matter. However, it is important to note that if detection were to in fact prevent transmission and mitigate risk, the system sensitivities calculated herein would had been underestimated.

Lastly, our calculated sample size was not adjusted for sampling with replacement. In large populations such adjustment is not needed, but considering the study example population of 816, for the 1% design prevalence strategy, a considerable proportion of the population would had been sampled (approximately 33%), which could have impacted the number of farms detected. Even though this was a limitation of the study, it should not influence comparison between surveillance strategies.

Future directions for prospective projects include the expansion of this tool for evaluation of different surveillance scenarios. For example, one could propose an active surveillance strategy based on monthly risk-based slaughterhouse sampling and evaluate how that compares to current methodologies, as well as assess the cost-benefit of such and other approaches. Finally, the mathematical modelling approach supports collaboration between multiple branches such as the private sector, swine veterinarians, academic researchers and governmental agencies, since input and feedback is needed from all involved parts to test new hypotheses and strategies that are relevant at the same time as logistically and economically viable to all mentioned parties. Eventually, the development of the methodology could be applied for other emerging pathogens, and within different regions of the country or country-wide.

## Conclusions

In conclusion, the model developed herein integrates the knowledge of the complex swine industry and characteristics of PRRSV transmission between herds to develop a comprehensive framework that could be used to test other hypotheses in the future regarding surveillance approaches for this and other emerging swine infectious diseases.

## Abbreviations

ABM: Agent-based model
ARC&E: Area Regional Control and Elimination
ODD: Overview, design concepts, and details
PRRS: Porcine reproductive and respiratory syndrome
PRRSV: Porcine reproductive and respiratory syndrome virus
STM: Scenario tree modelling
